# Salivary Lactate Dehydrogenase, Matrix Metalloproteinase-9, and Chemerin—The Most Promising Biomarkers for Oral Cancer? A Systematic Review with Meta-Analysis

**DOI:** 10.3390/ijms26167947

**Published:** 2025-08-18

**Authors:** Wojciech Owecki, Kacper Nijakowski

**Affiliations:** 1 Student’s Scientific Group in Department of Conservative Dentistry and Endodontics, Poznan University of Medical Sciences, 60-812 Poznan, Poland; 86897@student.ump.edu.pl; 2 The Student Scientific Society, Poznan University of Medical Sciences, 60-806 Poznan, Poland; 3 Department of Conservative Dentistry and Endodontics, Poznan University of Medical Sciences, 60-812 Poznan, Poland

**Keywords:** lactate dehydrogenase, matrix metalloproteinase-9, chemerin, oral cancer, biomarker, saliva

## Abstract

Oral cancer (OC) constitutes a significant health problem globally. There is an urgent need to develop novel biomarkers for OC diagnosis. This meta-analysis aimed to analyze the potential of salivary lactate dehydrogenase (LDH), matrix metalloproteinase-9 (MMP-9), and chemerin as OC biomarkers. The meta-analysis was conducted according to the PRISMA statement guidelines and registered in PROSPERO (CRD420251045968). PubMed, Embase, Scopus, and Web of Science databases were thoroughly searched up to 18 April 2025. After screening, thirty-three articles were included in the meta-analysis based on the random-effects model. The meta-analysis revealed a significantly elevated LDH level in OC patients compared with controls (SMD = 4.592, 95% CI: 3.580–5.605, *p* < 0.001) and with oral potentially malignant disorders (OPMD) (SMD = 2.416, 95% CI: 1.474–3.358, *p* < 0.001). For poorly versus well-differentiated OC, significantly higher LDH levels were observed in poorly differentiated tumors (SMD = 6.158, 95% CI: 0.739–11.576, *p* = 0.027). For MMP-9, there was a significant increase in OC compared with controls and a borderline-significant difference compared with OPMD (SMD = 1.507, 95% CI: 0.644–2.369, *p* = 0.001; SMD = 1.626, 95% CI: −0.097–3.350, *p* = 0.064, respectively). In comparing poorly versus well-differentiated OC, MMP-9 levels were significantly increased in poorly differentiated tumors (SMD = 1.790, 95% CI: 0.643–2.937, *p* = 0.003). Chemerin levels were significantly elevated in OC versus controls (SMD = 3.905, 95% CI: 3.210–4.600, *p* < 0.001) and OPMD (SMD = 1.605, 95% CI: 1.139–2.071, *p* < 0.001). In conclusion, these findings support the potential use of LDH, MMP-9, and chemerin as adjunctive biomarkers in diagnosing and stratifying OC.

## 1. Introduction

Oral cancer (OC) is one of the most common malignancies around the world [1]. Indeed, the incidence rates of OC are rising, and the evidence shows an increase of about 1% per year globally [2]. According to the National Cancer Institute, the overall 5-year survival rate in OC is around 68% [3]. Nevertheless, many patients are diagnosed in an advanced stage, which is associated with poor outcomes [4].

Furthermore, oncotherapy and oral health worsen the quality of life in OC patients [5]. Despite advances in OC therapy, the treatment measures for OC are expensive, and the affordability remains low, whereas the survival rates in OC have not improved in the past decades. Considering that, there is an urgent need to develop novel biomarkers for OC diagnosis and monitoring, as well as to employ preventive and screening methods to reduce the global burden of OC [6,7]. The latter aspect involves the detection of oral potentially malignant disorders (OPMD) associated with an increased risk of developing OC. These conditions include erythroplakia, leukoplakia (OL), oral submucous fibrosis (OSMF), lichen planus (LP), lichenoid lesions, actinic keratosis, and others [8]. On the other hand, developing novel, specific, and sensitive OC biomarkers may facilitate the early detection of malignant transformation into OC, monitoring disease progression and response to treatment.

One of the possible sources of biomarkers for OC diagnosis is saliva. This body fluid is gaining interest as a potential biofluid for non-invasive diagnostics of OC [9]. Human whole-mouth saliva comprises electrolytes, peptides, proteins, inorganic and organic salts secreted by salivary glands, as well as additional components from mucosal transudates and gingival crevicular fluids [10]. Saliva constitutes a valuable source of biomarkers since its collection is non-invasive, inexpensive, and straightforward, whereas saliva is durable and easily stored [11,12]. Indeed, studies show that saliva may be a diagnostic tool for multiple ailments, including cancers, cardiovascular, endocrinological, neurological, gastrointestinal, and other diseases [13,14,15,16,17,18,19,20,21,22].

Recent systematic reviews indicate that among the most promising salivary biomarkers for OC are lactate dehydrogenase (LDH), matrix metalloproteinase-9 (MMP-9), and chemerin [23,24]. LDH is a tetrameric enzyme mediating bidirectional pyruvate/lactate transformation [25]. Increased activity of LDH reflects inflammation, cellular damage and death, and may indicate malignant transformation [26]. Elevated levels of LDH are associated with poor outcomes in several malignancies, including neuroblastoma, cervical, and thyroid cancer [25]. On the other hand, MMP-9, a zinc-dependent proteolytic metalloenzyme, is involved in degrading components of the extracellular matrix. Dysregulation of MMP-9 is linked with various disorders, including inflammatory diseases or cancers [27,28]. In cancer pathology, MMP-9 induces the invasion of cancer cells, promoting tumor development [29]. Finally, chemerin is a multifunctional adipokine involved in glucose homeostasis, adipogenesis, inflammatory processes, and cancer pathomechanism [30]. Although the role of chemerin in cancer progression remains controversial, evidence shows that increased levels of circulating chemerin are associated with cancer risk [30,31,32].

Considering the beneficial aspects of saliva collection and promising results of LDH, MMP-9, and chemerin in OC detection, this meta-analysis aimed to investigate salivary levels of these three biomarkers in OC and assess their utility in OC diagnosis.

## 2. Results

### 2.1. Study Characteristics

**Figure 1:**
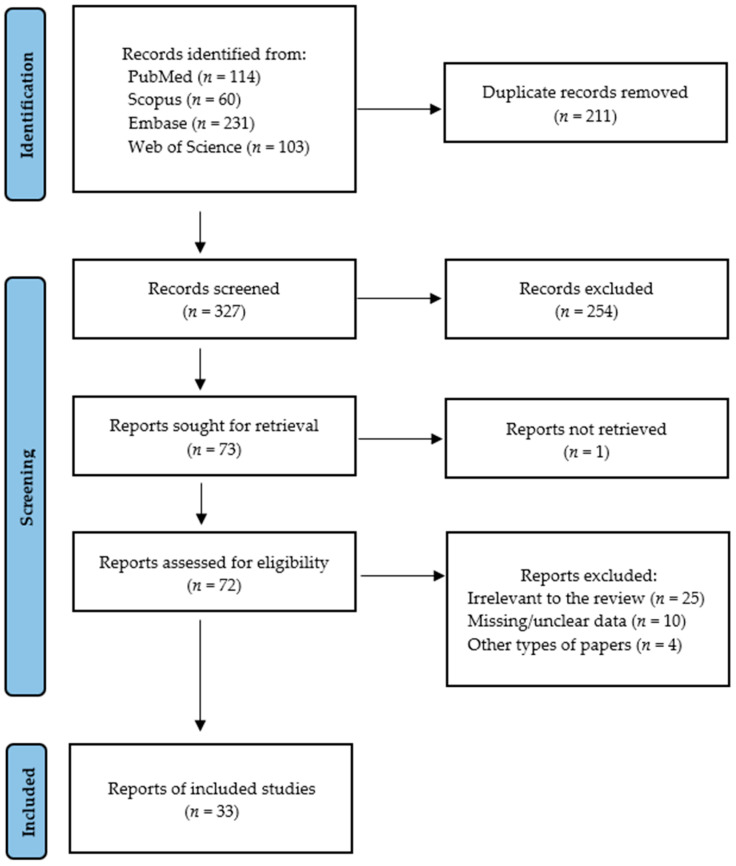
PRISMA flow diagram presenting search strategy.

Figure 1 presents the detailed selection strategy of the searched records. In Table 1, we demonstrated data regarding each eligible study included in this meta-analysis, which comprises the year of publication, setting, involved participants, OC or OSCC diagnosis, histological grading, type of saliva, centrifugation, storage, and method of biomarker determination.

In total, thirty-three articles were included in the meta-analysis. All studies were published between 2012 and 2024. Eligible studies recruited patients affected by OC. The majority of the included studies were conducted in India (22 studies, 66.67%), followed by Iran and Iraq (2 studies each, 6.06% each). Indeed, OC incidence in India is high and accounts for about 33% of the global cases [67]. The majority of the included studies investigated OSCC; however, three studies did not specify the OC type [44,45,49], one study recruited two patients with verrucous OC [65], and one study is unclear regarding this aspect [33]. Similarly, almost all the studies analyzed unstimulated saliva samples or the stimulation was not reported, except for two studies that utilized stimulated saliva [56,62] and one study investigating both types [40]. Centrifugation methods varied between groups, ranging from 900 rpm or 1000× *g* to 8000 rpm or 10,000× *g*. Storage temperatures also differed among studies, usually reaching −80 °C; however, higher temperatures were also reported (Mantri et al. [48] described storage at 4 °C). LDH, MMP-9, and chemerin levels were usually assessed using commercially available kits. The detailed characteristics of the included studies are presented in Table 1.

### 2.2. Quality Assessment

Figure S1 reports the summarized quality assessment. The most frequently encountered risks of bias were the absence of data regarding a clearly defined study population, group recruitment from the same population, randomization, and blinding. Critical appraisal was summarized by adding the points for each criterion of potential risk (points: 1—low, 0.5—unspecified, and 0—high). Twenty-five studies (75.8%) were classified as having a “good” quality (≥80% total score), and eight (24.2%) were classified as having an “intermediate” quality (≥60% total score).

All the included studies had a third or fourth level of evidence (case–control studies), according to the five-grade scale classification of the Oxford Centre for Evidence-Based Medicine levels for diagnosis [68].

### 2.3. Meta-Analysis

#### 2.3.1. Lactate Dehydrogenase (LDH)

**Figure 2:**
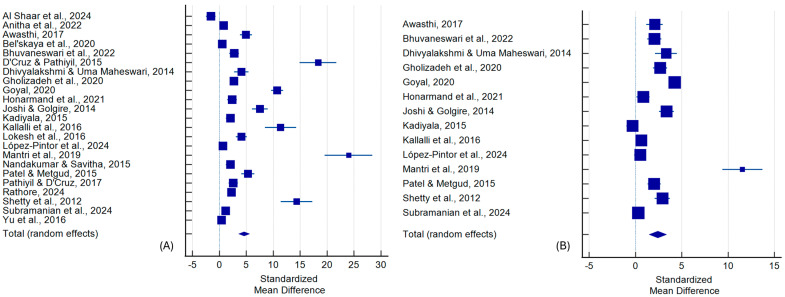
Forest plot with standardized mean differences comparing LDH levels between the following: (**A**) OC patients and healthy controls, (**B**) OC and OPMD patients [33,34,35,36,37,38,39,40,41,42,43,44,45,46,47,48,49,50,51,52,53,54,55].

Twenty-three studies were included in comparing LDH levels between OC and healthy controls. In total, eligible studies included 783 OC patients and 787 healthy controls. The random-effects meta-analysis revealed a significantly elevated LDH level in OC patients (SMD = 4.592, 95% CI: 3.580 to 5.605, *p* < 0.001), with substantial heterogeneity among studies (I^2^ = 97.81%). Egger’s and Begg’s tests indicated potential publication bias (*p* < 0.001 for both) (Figure 2A and Table 2).

Fourteen studies were included in the comparison between OC and OPMD. In total, eligible studies recruited 402 OC patients and 439 participants with OPMD. LDH levels remained significantly higher in OC (SMD = 2.416, 95% CI: 1.474 to 3.358, *p* < 0.001). Heterogeneity was again considerable (I^2^ = 96.05%), with borderline evidence of publication bias (Egger’s test *p* = 0.076; and Begg’s test *p* = 0.025) (Figure 2B, Table 2).

For poorly versus well-differentiated OC, the random-effects model (three studies: 19 patients with poorly differentiated and 38 patients with well-differentiated OC) showed a significant difference favoring higher LDH levels in poorly differentiated tumors (SMD = 6.158, 95% CI: 0.739 to 11.576, *p* = 0.027), although heterogeneity was high (I^2^ = 95.25%) (Figure S2A, Table 2).

#### 2.3.2. Matrix Metalloproteinase-9 (MMP-9)

**Figure 3:**
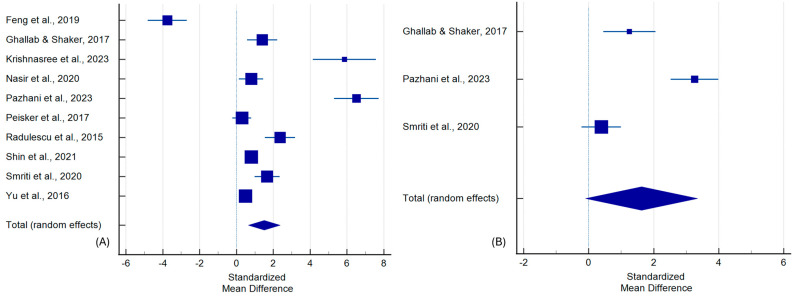
Forest plot with standardized mean differences comparing MMP-9 levels between the following: (**A**) OC patients and healthy controls, (**B**) OC and OPMD patients [55,56,57,58,59,60,61,62,63,64,65].

Ten studies compared MMP-9 levels between OC and controls, encompassing 465 OC patients and 521 healthy controls. The pooled random-effects SMD was 1.507 (95% CI: 0.644 to 2.369, *p* = 0.001), indicating a significant increase in OC. Heterogeneity was substantial (I^2^ = 96.15%), with evidence of publication bias by Begg’s test (*p* = 0.040) (Figure 3A, Table 2).

Three studies were included to compare OC and OPMD, encompassing 73 OC patients and 69 participants with OPMD. The random-effects model showed an elevated borderline-significant difference (SMD = 1.626, 95% CI: −0.097 to 3.350, *p* = 0.064), and heterogeneity remained high (I^2^ = 94.59%) (Figure 3B, Table 2).

In the comparison of poorly versus well-differentiated OC (four studies; 28 patients with poorly differentiated OC and 51 patients with well-differentiated OC), MMP-9 levels were significantly higher in poorly differentiated tumors (SMD = 1.790, 95% CI: 0.643 to 2.937, *p* = 0.003), with high heterogeneity (I^2^ = 75.93%) (Figure S2B, Table 2).

#### 2.3.3. Chemerin

**Figure 4:**
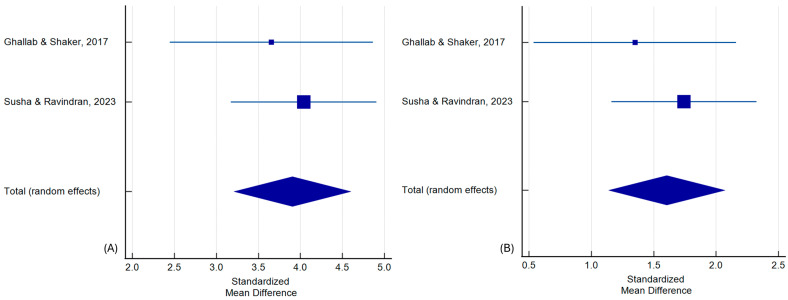
Forest plot with standardized mean differences comparing chemerin levels between the following: (**A**) OC patients and healthy controls, (**B**) OC and OPMD patients [57,66].

Two studies assessed chemerin levels in OC vs. controls, with consistent findings of significantly higher levels in OC (random-effects SMD = 3.905, 95% CI: 3.210 to 4.600, *p* < 0.001). There was no heterogeneity (I^2^ = 0%) (Figure 4A, Table 2).

Similarly, chemerin levels were significantly elevated in OC compared to OPMD (SMD = 1.605, 95% CI: 1.139 to 2.071, *p* < 0.001), also with no observed heterogeneity (I^2^ = 0%) (Figure 4B, Table 2).

For both analyses, Egger’s test suggested potential publication bias (*p* < 0.001). In total, two studies recruited 47 OC patients, 47 participants with OPMD, and 47 healthy controls.

## 3. Discussion

In this study, we performed a meta-analysis regarding three promising salivary biomarkers for OC detection: LDH, MMP-9, and chemerin. These particular biomarkers were selected based on recently published systematic reviews and meta-analyses. A 2024-published systematic review (without any meta-analyses) concluded that the most promising biomarkers for saliva-based OC diagnosis are TNF-α, IL-1β, IL-6, IL-8, LDH, and MMP-9 [23]. A recent meta-analysis by Huang et al. [69] compared salivary interleukins and TNF-α levels in OC patients and healthy controls. TNF-α seemed to be the most precise biomarker for OC diagnosis (sensitivity: 79%, specificity: 92%), followed by IL-6 (sensitivity: 75%, specificity: 86%), IL-8 (sensitivity: 80%, specificity: 80%), and IL-1β (sensitivity: 66%, specificity: 75%) [69]. The latter study was preceded by a similar meta-analysis analyzing salivary cytokines in OC detection, published in 2021 [70]. On the other hand, another recent network meta-analysis [24] indicated that salivary chemerin and MMP-9 are the top biomarkers in early OSCC, having the highest sensitivity and balanced accuracy. However, this network meta-analysis was based on four earlier meta-analyses published in or before 2021 [24]. Considering that diagnostic capabilities in oncology are rapidly evolving and the research is ongoing, we decided to include chemerin and MMP-9 in the current meta-analysis. Moreover, our meta-analysis investigated LDH, which was not discussed in the above-mentioned meta-analyses [24,69,70].

Growing evidence suggests LDH implication in cancer development [71]. Most cancer cells have abnormal metabolism with the promotion of aerobic glycolysis and lactate production, as well as increased glucose uptake [72]. LDH plays a key role in this process, catalyzing the inter-conversion between pyruvate and lactate [73,74]. Indeed, excessive levels of lactate induce extracellular acidosis, affecting the immune response and facilitating tumor invasion, angiogenesis, and metastasis. Furthermore, lactate symbiosis and lactate shuttle in the tumor cells contribute to poor prognosis [75]. Besides LDH being involved in cancer cell metabolism and adaptation to unfavorable conditions, this enzyme is also implicated in regulating cell death [76]. Reports show that LDH is associated with multiple types of cancers, such as pancreatic, breast, colorectal, and lung cancer [74,77,78,79]. Evidence confirms that LDH is also involved in OSCC development. LDHA, an LDH isoenzyme with the highest affinity for pyruvate/lactate conversion, acts as an oncogene, inducing malignant OSCC progression via promoting glycolysis and epithelial–mesenchymal transition [74,80]. Indeed, this meta-analysis demonstrates that LDH levels in saliva significantly increase in OC patients. Interestingly, similar observations were found in the serum of OC patients [40,54]. Moreover, a 2022-published meta-analysis revealed that elevated serum levels of LDH are significantly associated with OPMD, which may precede OC development [81]. Our meta-analysis shows that salivary LDH is significantly higher in OC than in OPMD. These findings are consistent with findings from a meta-analysis by Iglesias-Velázquez [82], encompassing studies published until 2020. Moreover, our results indicate that salivary LDH levels are significantly higher in poorly differentiated OC compared with well-differentiated OC, suggesting that salivary LDH may serve as a diagnostic and prognostic OC biomarker.

Reports indicate that MMP-9 is also involved in cancer pathogenesis [29]. For instance, MMP-9 modulates the dynamic remodeling of extracellular matrix (affecting collagens, aggrecan, fibronectin, elastin, glycosaminoglycans, laminins, and latent signaling proteins) by proteolytic cleavages, releasing factors that alter cellular regulation [83]. Moreover, MMP-9 is involved in basement membrane destruction. Importantly, basement membrane degradation is often essential in cancer development, supporting tumor invasion and metastases [28]. Furthermore, MMP-9 induces endothelial cell migration and activates the angiogenic switch via increased vascular endothelial growth factor (VEGF) release during cancer development [83]. Additionally, reports indicate that MMP-9 knockdown may reduce cancer invasion and metastasis [84,85]. Interestingly, in thyroid cancer, MMP-9 may induce tumor invasion by promoting epithelial–mesenchymal transition, thus altering the migration and invasion ability of cancer cells [86]. Concomitantly, MMP-9 may also have an antagonistic effect, inhibiting angiogenesis by cleaving plasminogen and producing angiostatin molecules [87]. The role of MMP-9 in OSCC pathogenesis, OSCC invasion, and metastasis seems to be fluctuating, as discussed in detail in another paper [88]. Nevertheless, meta-analyses show that the increased expression of MMP-9 in OC is correlated with clinical stage and poor outcome in OC patients, and that MMP-9 overexpression may serve as a prognostic biomarker in OC [89,90]. Indeed, the results of our meta-analysis indicate a significant increase in salivary MMP-9 in OC compared with controls and a borderline-significant increase compared with OPMD. In comparing poorly versus well-differentiated OC, MMP-9 levels were significantly increased in poorly differentiated tumors, suggesting that MMP-9 may play a prognostic role in OC.

Chemerin is a relatively newly described molecule whose role in cancer development remains unclear [30,91]. Chemerin may promote tumorigenesis by recruiting tumor-supporting mesenchymal stromal cells and modulating proangiogenic pathways in endothelial cells [92]. Moreover, chemerin influences the phosphorylation of p42-p44 MAP kinases or the recruitment of β-arrestin 1 and 2 to G-protein coupled receptor 1 (GPR1) or chemokine-like receptor 1 (CMKLR1). These proteins are implicated in cancer development; however, with diverse effects: β-arrestin 1 induces cancer growth, whereas β-arrestin 2 prevents angiogenesis and tumor growth [93,94]. Interestingly, chemerin may interact with MMPs, activating them and stimulating cancer cell invasion and metastasis. Nevertheless, the evidence is inconsistent; in breast cancer, the opposite effect was observed [94]. In OC (OSCC of the tongue, precisely), a study by Wang et al. [95] revealed that chemerin was overexpressed in OSCC tissue. Moreover, reports show that chemerin is associated with tumor angiogenesis, metastasis, and poor clinical outcomes in OSCC patients [95,96]. Furthermore, chemerin may induce neutrophil infiltration in OSCC by upregulating chemokines CXCL-5 and IL-17 or by activating the MEK/ERK signaling pathway [97,98]. Additionally, chemerin facilitates OSCC invasion by stimulating TNF-α and IL-6 synthesis via STAT3 activation [99]. Indeed, the results of our meta-analysis show that salivary chemerin is significantly elevated in OC patients compared with healthy controls and participants with OPMD, suggesting that it may serve as a potential OC biomarker.

Interestingly, studies included in this meta-analysis highlighted some important aspects. Gholizadeh et al. [40] investigated LDH levels in the unstimulated and stimulated saliva of participants with LP, lichenoid reactions, OSCC patients, and healthy controls. LDH levels were increased in the stimulated saliva of OSCC and LR patients compared with unstimulated samples. In contrast, in healthy controls and participants with LP, LDH levels were decreased in stimulated saliva compared with unstimulated samples. Another study analyzed five isoenzymes of LDH and concluded that the levels of three isoenzymes were significantly elevated, one showed no difference, and one was insignificantly decreased in the OSCC group compared with controls [43].

On the other hand, some studies noticed a general tendency of lower LDH levels in females compared to males, whereas other articles indicated opposite results [44,47,49,53]. Moreover, Pathiyil et al. [51] suggested a prognostic utility of salivary LDH, confirming a significant decrease in salivary LDH level one month after surgery in OSCC patients. Similar findings were described for MMP-9, although one study showed an insignificant decrease. In contrast, other research found a significant decrease in MMP-9 only three months after surgery, with insignificant values for longer follow-up after operation [59,64]. Additionally, Nisa et al. [60] demonstrated a significant increase in salivary MMP-9 along with OSCC duration. In contrast, Peisker et al. [62] concluded that salivary MMP-9 is not useful for detecting OSCC recurrence in the follow-up, since there were no significant differences in comparison between healthy participants and OSCC patients with recurrence.

### Study Limitations and Future Directions

This study has some limitations. The included studies were highly heterogeneous; however, subgroup analysis could not be implemented, as the included studies were predominantly limited to unstimulated saliva and specific detection methods as well as originated from a single geographic region, mainly Asia. It should also be noted that only two of the included studies investigated chemerin, which restricts the generalizability of the findings. Some studies were not classified as having a good quality and lacked the detailed reporting of specific information. Another limitation of this meta-analysis is the exclusion of studies not published in English. The results of Egger’s and Begg’s tests indicate potential publication bias, particularly for LDH and chemerin. Such bias can lead to an overestimation of the true effect sizes in meta-analyses, as the pooled estimates become skewed by disproportionately favorable outcomes. Moreover, minimal reporting of the results of ROC analysis to assess the predictive reliability of biomarkers also limited this meta-analysis.

Future studies should prioritize rigorous experimental design, with particular attention to standardizing pre-analytical variables such as storage temperature, time of sample collection, whether saliva is stimulated or unstimulated, and the specific processing methods used. These factors can significantly influence biomarker stability and reproducibility, and their lack of consistency has been a major limitation in the field. Moreover, comparative analyses with blood—the current gold standard—are essential to validate the reliability and diagnostic value of salivary biomarkers.

## 4. Materials and Methods

### 4.1. Search Strategy and Data Extraction

Our meta-analysis was conducted based on a systematic review of records published from database inception to 18 April 2025, according to the Preferred Reporting Items for Systematic Reviews and Meta-Analyses (PRISMA) statement guidelines [100], using the databases PubMed, Embase, Scopus and Web of Science. The search queries included the following:

- for PubMed: ((LDH OR Lactate dehydrogenase) OR (chemerin) OR (MMP-9 OR matrix metalloproteinase-9)) AND saliva* AND (oral cancer OR oral carcinoma OR oral squamous cell carcinoma OR oscc);
- for Embase: ((LDH OR Lactate dehydrogenase) OR (chemerin) OR (MMP-9 OR matrix metalloproteinase-9)) AND saliva* AND (oral cancer OR oral carcinoma OR oral squamous cell carcinoma OR oscc);
- for Scopus: TITLE-ABS-KEY ((LDH OR Lactate dehydrogenase) OR (chemerin) OR (MMP-9 OR matrix metalloproteinase-9)) AND saliva* AND (oral cancer OR oral carcinoma OR oral squamous cell carcinoma OR oscc);
- for Web of Science: TS = ((LDH OR Lactate dehydrogenase) OR (chemerin) OR (MMP-9 OR matrix metalloproteinase-9)) AND saliva* AND (oral cancer OR oral carcinoma OR oral squamous cell carcinoma OR oscc).

Records were screened by the title, abstract, and full text and were analyzed by two independent investigators. Studies included in this review matched all the predefined criteria according to PI(E)COS (“Population”, “Intervention”/”Exposure”, “Comparison”, “Outcomes”, and “Study design”), as shown in Table 3. A detailed search flowchart is presented in Section 2. The study protocol was registered in the International prospective register of systematic reviews PROSPERO (CRD420251045968).

The results of the meta-analysis were presented in forest plots using the MedCalc Statistical Software, version 22.014 (MedCalc Software Ltd., Ostend, Belgium). The meta-analysis was performed for salivary LDH, MMP-9, and chemerin. The standardized mean differences were calculated.

### 4.2. Quality Assessment of Included Studies

The risk of bias in each individual study was assessed according to the “Study Quality Assessment Tool” issued by the National Heart, Lung, and Blood Institute within the National Institute of Health [101]. These questionnaires were answered by two independent investigators, and any disagreements were resolved by discussion between them.

## 5. Conclusions

This meta-analysis demonstrates that LDH, MMP-9, and chemerin are significantly elevated in patients with OC compared to both healthy controls and individuals with OPMD. Notably, LDH showed the largest effect sizes across all comparisons, suggesting it may serve as a particularly robust biomarker for OC detection. However, the marked heterogeneity and evidence of publication bias, especially for LDH and MMP-9, highlight the need for cautious interpretation and further standardized studies.

Chemerin emerged as a consistent and promising marker with large effect sizes and no heterogeneity, indicating strong reproducibility and potential utility in distinguishing OC from both healthy tissue and OPMD. Furthermore, both LDH and MMP-9 levels were significantly higher in poorly differentiated OC than well-differentiated tumors, supporting their role in diagnosis and prognostic assessment.

These findings support the potential use of LDH, MMP-9, and chemerin as adjunctive biomarkers in the diagnosis and stratification of OC. Future research should aim to validate these markers in larger, prospective cohorts, and explore their integration into clinical screening protocols.

## Figures and Tables

**Table 1:** Characteristics of included studies.

Author, Year	Setting	Study Group—OC; (F/M), Age	Study Group—OPMD; (F/M), Age	Control Group; (F/M), Age	Diagnosis	Histological Grading	Type of Saliva	Centrifugation and Storing	Method of Marker Determination
**LDH**									
Al Shaar et al., 2024 [33]	Syria	12; (6/60), 57.67 ± 13.98	LP: 15; (8/7), 46.13 ± 14.08	15; (7/8), 24.4 ± 2.95	OC/OSCC	NR	unstimulated	centrifuged at 3000 rpm for 3 min; NR	Hitachi 911 automated clinical chemistry analyzer
Anitha et al., 2022 [34]	India	18; (2/16), 44.67	-	18; (5/13), 34.56	OSCC	MD: 13 WD: 5	unstimulated	centrifuged at 2000 rpm for 10 min, stored at −20 °C	ErbaCHEM 5× semi-automatic analyzer machine, LDH-P reagent kit
Awasthi et al., 2017 [35]	India	30; (2/28), 49.6 (25–70)	9; (1/8), 34.2 (25–40)	25; (3/22), 48.1 (25–68)	OSCC	PD: 1 MD: 20 WD: 9	unstimulated	centrifuged at 3000 rpm for 15 min, stored at −80 °C	standard kit method
Bel’skaya et al., 2020 [36]	Russia	68; NR, NR	-	114; NR, NR	OSCC	NR	NR	centrifuged at 10,000× *g* for 10 min, no storage	kinetic ultraviolet method according to the NADH (Nicotinamide Adenine Dinucleotide) oxidation rate
Bhuvaneswari et al., 2022 [37]	India	21; NR, NR	OL: 20; NR, NR	20; NR, NR	OSCC	NR	unstimulated	centrifuged at “1000 rotations” at 4 °C for 10 min, stored at −80 °C	LDH enzyme kit, ultraviolet-visible spectrophotometer
D’Cruz et al., 2015 [38]	India	30; NR, NR	-	30; NR, NR	OSCC	PD: 10 MD: 10 WD: 10	unstimulated	NR	standard kit, measured spectrophotometrically at 340 nm
Dhivyalakshmi et al., 2014 [39]	India	14; NR, NR	OL: 14; NR, NR	14; NR, NR	OSCC	NR	unstimulated	centrifuged at 2500 rpm for 15 min, NR	standard kit, measured using autoanalyzer
Gholizadeh et al., 2020 [40]	Iran	25; (15/10), 61.00 ± 3.23	LP: 15; (17/8), 49.73 ± 3.19; LR: 25; (17/8), 52.73 ± 2.78	25; (17/8), 42.73 ± 2.38	OSCC	NR	unstimulated and stimulated	centrifuged at 2000 rpm for 10 min, stored at −20 °C	spectrophotometrically measured within 24 h, standard LDH kits
Goyal et al., 2020 [41]	India	100; NR, NR	100; NR, NR	100; NR, NR	OSCC	NR	unstimulated	centrifuged at 2500 rpm for 15 min, NR	standard kit method
Honarmand et al., 2021 [42]	Iran	15; NR, 50.4 ± 8.37	LP: 20; NR, 45.4 ± 10.08	20; NR, 45.6 ± 9.77	OSCC	NR	unstimulated	centrifuged at 3500 rpm for 20 min, stored at −70 °C	ELISA
Joshi et al., 2014 [43]	India	30; (10/20), 47.96	OL: 30; (1/29), 41.06	30; NR, NR	OSCC	PD: 1 MD: 7 WD: 22	unstimulated	centrifuged at 1000 rpm for 10 min, NR	agarose gel electrophoresis method (SEBIA-HYDRAGEL ISO-LDH K-20 kit)
Kadiyala et al., 2015 [44]	India	20; NR, NR	OSMF: 20; NR, NR	20; NR, NR	OC	NR	unstimulated	centrifuged at 2500 rpm for 15 min, NR	ERBA CHEM 5 semi-automatic analyzer
Kallalli et al., 2016 [45]	India	25; NR, NR	OSMF: 25; NR, NR	10; NR, NR	OC	NR	unstimulated	centrifuged NR, NR	ERBA-CHEM 5 semi-automatic analyzer
Lokesh et al., 2016 [46]	India	30; NR, 35–65	-	20; NR, NR	OSCC	PD: 5 MD: 10 WD: 15	unstimulated	centrifuged NR, no storage	automated method using autoanalyzer readings, spectrophotometer at a wavelength of 340 nm (UV kinetic method)
López-Pintor et al., 2024 [47]	Spain	12; (8/4), 69 ± 12.87	51; (35/16), 64.65 ± 10.39	29; (17/12), 59.83 ± 13.82	OSCC	PD: 1 MD: 3 WD: 8	unstimulated	centrifuged at 1160× *g* for 20 min, stored at −80 °C	LDH Assay Kit Colorimetric analyzed spectrophotometrically at a wavelength of 450 nm
Mantri et al., 2019 [48]	India	30; NR, NR	OSMF: 30; NR, NR	30; NR, NR	OSCC	NR	unstimulated	centrifuged at 5000 rpm for 5 min, stored at 4 °C	LDH-P kit within 24 h, analyzed by an Erba Chem UV semi-automated spectrophotometer
Nandakumar et al., 2015 [49]	India	20; (8/12), female: 37.50 ± 5.01; male: 40.83 ± 4.35	-	20; (2/18), female: 41.00 ± 2.82; male: 39.56 ± 4.50	OC	NR	unstimulated	centrifuged at 2500 rpm for 15 min, NR	ERBA CHEM 5 semi-automatic analyzer
Patel et al., 2015 [50]	India	25; NR, NR	OL: 25; NR, NR	25; NR, NR	OSCC	PD: 4 MD: 8 WD: 13	unstimulated	NR, stored in an ice box	Semi-automatic Analyzer by using Biovision LDH Activity Colorimetric Assay Kit
Pathiyil et al., 2017 [51]	India	20; NR, NR	-	20; NR, NR	OSCC	NR	unstimulated	centrifuged at 3000 rpm for 10 min, NR	standard kit, measured spectrophotometrically at 340 nm
Rathore et al., 2024 [52]	India	54; (16/38) NR	-	54; NR, NR	OSCC	NR	unstimulated	centrifuged at 3000 rpm for 15 min, NR	standard kit method
Shetty et al., 2012 [53]	India	25; NR, NR	OL: 25; NR, NR	25; NR, NR	OSCC	NR	unstimulated	NR	standard kit, measured sphectrophotometrically at 340 nm
Subramanian et al., 2024 [54]	India	30; (14/16), NR	30; (6/24), NR	30; (18/12), NR	OSCC	NR	unstimulated	centrifuged at 900 rpm for 12 min, stored at −20°C	LDH kit (Liquizyme), semi-automatic analyzer (spectrophotometer)
Yu et al., 2016 [55]	Taiwan	131; (2/129), 52.5 ± 9.7; detectable: 129	103; (1/102), 49.5 ± 10.7	96; (0/96), 48.8 ± 11.8; detectable: 93	OSCC	NR	unstimulated	centrifuged at 3000× *g* for 15 min at 4 °C, stored at −80°C	Liquid Chromatography-multiple reaction monitoring-Mass Spectrometry
**MMP-9**									
Feng et al., 2019 [56]	China	20; NR, NR	-	20; NR, NR	OSCC	NR	stimulated	centrifuged at 10,000× *g* for 10 min at 4°C, stored at −80 °C	Human Protease Array Kit, human protease ELISA kits
Ghallab et al., 2017 [57]	Egypt	15; (9/6), 47.66 ± 14.07	15; (8/7), 42.33 ± 10.99	15; (9/6), 43.26 ± 11.82	OSCC	NR	unstimulated	centrifuged at 10,000× *g* for 2 min, stored at −80 °C	Quantikine ELISA kit
Krishnasree et al., 2023 [58]	India	15; (NR), 64 ± 4	-	15; (NR), 60 ± 3.5	OSCC	PD: 2 MD: 4 WD: 9	unstimulated	centrifuged NR, stored at −80 °C	MMP-9 ELISA kit
Nasir et al., 2020 [59]	Iraq	Before treatment: 20; (NR), NR After treatment: 20; (NR), NR	-	20; (NR), NR	OSCC	NR	unstimulated	NR	MMP-9 ELISA kit
Nisa et al., 2023 [60]	Pakistan	45; (10/35), 18–70	-	45; (18/27), NR	OSCC	PD: 15 MD: 15 WD: 15	NR	centrifuged at 8000 rpm for 15 min at 4 °C, stored at −80 °C	ELISA Bioassay Technology kit
Pazhani et al., 2023 [61]	India	34; (6/28), 62.8 ± 12.9	OL: 34; (11/23), 60.1 ± 11.5	34; (22/12), 52.4 ± 9.7	OSCC	PD: 5 MD: 9 WD: 20	unstimulated	centrifuged NR, stored at −80 °C	MMP-9 ELISA kit
Peisker et al., 2017 [62]	Germany	30; (16/14), 65.0 ± 10.9	-	30; (12/18), 60.7 ± 12.3	OSCC	UD: 1 PD: 8 MD: 20 WD: 1	stimulated	centrifuged at 1000× *g* for 2 min at 20 °C, NR	ELISA
Radulescu et al., 2015 [63]	Romania	30; (16/14), 45–60	-	14; (NR), 40–60	OSCC	NR	unstimulated	centrifuged at 3000 rpm for 10 min, stored at −80 °C	MMP-9 ELISA kit
Shin et al., 2021 [64]	South Korea	106; (44/62), 63.14 ± 9.7	-	212; (88/124), 63.09 ± 9.7	OSCC	NR	unstimulated	centrifuged at 2600 rpm for 15 min at 4 °C, stored at −80 °C	Quantikine1 human MMP-9 immunoassay ELISA kit
Smriti et al., 2020 [65]	India	24; (10/14), 58.63 ± 14.79	20; (6/14), 44 ± 14.19	22; (7/15), 48.09 ± 11.73	OSCC/verrucous OC (2 patients)	PD: 6 MD: 9 WD: 7	unstimulated	centrifuged at 4000× *g* for 10 min at 4°C, NR	Human MMP-9 PicokineTM ELISA kit
Yu et al., 2016 [55]	Taiwan	131; (2/129), 52.5 ± 9.7; detectable: 126	103; (1/102), 49.5 ± 10.7	96; (0/96), 48.8 ± 11.8; detectable: 94	OSCC	NR	unstimulated	centrifuged at 3000× *g* for 15 min at 4 °C, stored at −80 °C	Liquid Chromatography-multiple reaction monitoring-Mass Spectrometry
**CHEMERIN**									
Susha et al., 2023 [66]	India	32; (6/28), 31–40: 3; 41–50: 3; 51–60: 8; 61–70: 9; >70: 9	OL: 32; (NR), NR	32; (NR), NR	OSCC	PD: 4 MD: 8 WD: 20	unstimulated	centrifuged at 3000 rpm for 10 min, stored at −80 °C	ab155430 Chemerin Human ELISA kit
Ghallab et al., 2017 [57]	Egypt	15; (9/6), 47.66 ± 14.07	15; (8/7), 42.33 ± 10.99	15; (9/6), 43.26 ± 11.82	OSCC	NR	unstimulated	centrifuged at 10,000× *g* for 2 min, stored at −80 °C	RD191136200R Human Chemerin ELISA

Abbreviations: ELISA, enzyme-linked immunosorbent assay; LDH, lactate dehydrogenase; LP, lichen planus; LR, lichenoid reaction; MD, moderately differentiated; MMP-9, matrix metalloproteinase-9; NR, not reported; OC, oral cancer; OL, oral leukoplakia; OPMD, oral potentially malignant disorders; OSCC, oral squamous cell carcinoma; OSMF, oral submucosal fibrosis; PD, poorly differentiated; UD, undifferentiated; WD, well differentiated.

**Table 2:** Detailed results of the performed meta-analyses.

Study	SMD	SE	95% CI	*p*-Value	Weight%
**OC Patients vs. Healthy Controls**					
*LDH*					
Al Shaar et al., 2024 [33]	−1.559	0.431	−2.447 to −0.671		4.61
Anitha et al., 2022 [34]	0.811	0.340	0.121 to 1.501		4.66
Awasthi et al., 2017 [35]	4.958	0.543	3.869 to 6.047		4.52
Bel’skaya et al., 2020 [36]	0.520	0.155	0.214 to 0.825		4.74
Bhuvaneswari et al., 2022 [37]	2.742	0.436	1.859 to 3.625		4.60
D’Cruz & Pathiyil, 2015 [38]	18.323	1.692	14.936 to 21.710		3.15
Dhivyalakshmi & Uma Maheswari, 2014 [39]	4.093	0.659	2.739 to 5.447		4.42
Gholizadeh et al., 2020 [40]	2.708	0.388	1.927 to 3.489		4.63
Goyal et al., 2020 [41]	10.747	0.556	9.652 to 11.843		4.51
Honarmand et al., 2021 [42]	2.364	0.437	1.474 to 3.253		4.60
Joshi & Golgire, 2014 [43]	7.531	0.733	6.063 to 8.999		4.34
Kadiyala et al., 2015 [44]	2.040	0.385	1.261 to 2.819		4.64
Kallalli et al., 2016 [45]	11.385	1.409	8.518 to 14.251		3.51
Lokesh et al., 2016 [46]	4.087	0.498	3.086 to 5.087		4.56
López-Pintor et al., 2024 [47]	0.646	0.344	−0.050 to 1.342		4.66
Mantri et al., 2019 [48]	24.000	2.206	19.585 to 28.415		2.55
Nandakumar & Savitha, 2015 [49]	2.040	0.385	1.261 to 2.819		4.64
Patel & Metgud, 2015 [50]	5.308	0.599	4.103 to 6.513		4.47
Pathiyil & D’Cruz, 2017 [51]	2.579	0.423	1.722 to 3.436		4.61
Rathore et al., 2024 [52]	2.255	0.245	1.769 to 2.741		4.71
Shetty et al., 2012 [53]	14.352	1.462	11.412 to 17.291		3.44
Subramanian et al., 2024 [54]	1.180	0.277	0.626 to 1.734		4.69
Yu et al., 2016 [55]	0.395	0.137	0.125 to 0.665		4.74
Total (random effects)	4.592	0.516	3.580 to 5.605	<0.001	
Egger’s test				<0.001	
Begg’s test				<0.001	
*MMP-9*					
Feng et al., 2019 [56]	−3.754	0.522	−4.810 to −2.698		9.55
Ghallab & Shaker, 2017 [57]	1.408	0.399	0.590 to 2.225		10.11
Krishnasree et al., 2023 [58]	5.853	0.835	4.143 to 7.564		7.89
Nasir et al., 2020 [59]	0.796	0.322	0.144 to 1.449		10.41
Pazhani et al., 2023 [61]	6.510	0.608	5.297 to 7.723		9.11
Peisker et al., 2017 [62]	0.300	0.256	−0.213 to 0.813		10.63
Radulescu et al., 2015 [63]	2.363	0.406	1.544 to 3.181		10.08
Shin et al., 2021 [64]	0.802	0.123	0.561 to 1.044		10.94
Smriti et al., 2020 [65]	1.667	0.338	0.986 to 2.349		10.36
Yu et al., 2016 [55]	0.496	0.138	0.224 to 0.768		10.91
Total (random effects)	1.507	0.439	0.644 to 2.369	0.001	
Egger’s test				0.279	
Begg’s test				0.040	
*Chemerin*					
Ghallab & Shaker, 2017 [57]	3.655	0.591	2.446 to 4.865		35.07
Susha & Ravindran, 2023 [66]	4.040	0.434	3.172 to 4.908		64.93
Total (random effects)	3.905	0.350	3.210 to 4.600	<0.001	
Egger’s test				<0.001	
Begg’s test				0.317	
**OC vs. OPMD patients**					
*LDH*					
Awasthi, 2017 [35]	2.074	0.440	1.182 to 2.966		7.14
Bhuvaneswari et al., 2022 [37]	2.058	0.381	1.286 to 2.830		7.25
Dhivyalakshmi & Uma Maheswari, 2014 [39]	3.313	0.575	2.131 to 4.495		6.85
Gholizadeh et al., 2020 [40]	2.678	0.386	1.901 to 3.454		7.24
Goyal et al., 2020 [41]	4.253	0.255	3.750 to 4.756		7.44
Honarmand et al., 2021 [42]	0.836	0.348	0.128 to 1.545		7.31
Joshi & Golgire, 2014 [43]	3.368	0.399	2.569 to 4.167		7.22
Kadiyala et al., 2015 [44]	−0.320	0.312	−0.952 to 0.312		7.36
Kallalli et al., 2016 [45]	0.632	0.285	0.058 to 1.206		7.40
López-Pintor et al., 2024 [47]	0.530	0.320	−0.110 to 1.171		7.35
Mantri et al., 2019 [48]	11.563	1.086	9.389 to 13.736		5.47
Patel & Metgud, 2015 [50]	2.036	0.345	1.343 to 2.730		7.31
Shetty et al., 2012 [53]	2.912	0.403	2.102 to 3.722		7.21
Subramanian et al., 2024 [54]	0.319	0.256	−0.195 to 0.832		7.44
Total (random effects)	2.416	0.480	1.474 to 3.358	<0.001	
Egger’s test				0.076	
Begg’s test				0.025	
*MMP-9*					
Ghallab & Shaker, 2017 [57]	1.254	0.390	0.454 to 2.054		32.95
Pazhani et al., 2023 [61]	3.260	0.368	2.525 to 3.996		33.19
Smriti et al., 2020 [65]	0.387	0.300	−0.218 to 0.993		33.86
Total (random effects)	1.626	0.872	−0.097 to 3.350	0.064	
Egger’s test				0.554	
Begg’s test				0.602	
*Chemerin*					
Ghallab & Shaker, 2017 [57]	1.350	0.396	0.539 to 2.160		35.11
Susha & Ravindran, 2023 [66]	1.743	0.291	1.161 to 2.325		64.89
Total (random effects)	1.605	0.234	1.139 to 2.071	<0.001	
Egger’s test				<0.001	
Begg’s test				0.317	
**Poorly and well-differentiated OC patients**					
*LDH*					
D’Cruz & Pathiyil, 2015 [38]	14.282	2.298	9.453 to 19.110		28.93
Lokesh et al., 2016 [46]	4.930	0.923	2.991 to 6.870		35.07
Patel & Metgud, 2015 [50]	0.827	0.561	−0.369 to 2.022		36.00
Total (random effects)	6.158	2.704	0.739 to 11.576	0.027	
Egger’s test				0.113	
Begg’s test				0.117	
*MMP-9*					
Krishnasree et al., 2023 [58]	1.264	0.637	−0.205 to 2.733		23.82
Nisa et al., 2023 [60]	1.137	0.384	0.350 to 1.925		29.23
Pazhani et al., 2023 [61]	3.972	0.741	2.439 to 5.506		21.59
Smriti et al., 2020 [65]	1.178	0.567	−0.070 to 2.425		25.36
Total (random effects)	1.790	0.576	0.643 to 2.937	0.003	
Egger’s test				0.316	
Begg’s test				0.042	

**Table 3:** Inclusion and exclusion criteria according to the PECOS.

Parameter	Inclusion Criteria	Exclusion Criteria
Population	Patients aged 0–99 years, both genders	-
Exposure	Oral cancer	Cancers other than oral cancer, head and neck cancer without precise localization
Comparison	Healthy subjects	-
Outcomes	Salivary LDH, MMP-9, chemerin	Other salivary alterations
Study design	Case–control, cohort, and cross-sectional studies	Literature reviews, case reports, expert opinion, letters to the editor, conference reports
	Indexed to 18 April 2025	Not published in English

## Data Availability

Data are available on request from the corresponding author.

## Supplementary Materials

The supporting information can be downloaded at: https://www.mdpi.com/article/10.3390/ijms26167947/s1.

## References

[1] Montero P.H., Patel S.G. (2015). Cancer of the Oral Cavity. Surg. Oncol. Clin. N. Am..

[2] Siegel R.L., Kratzer T.B., Giaquinto A.N., Sung H., Jemal A. (2025). Cancer Statistics, 2025. CA Cancer J. Clin..

[3] Oral Cancer 5-Year Survival Rates|Data & Statistics|National Institute of Dental and Craniofacial Research. https://www.nidcr.nih.gov/research/data-statistics/oral-cancer/survival-rates.

[4] Gormley M., Gray E., Richards C., Gormley A., Richmond R.C., Vincent E.E., Dudding T., Ness A.R., Thomas S.J. (2022). An Update on Oral Cavity Cancer: Epidemiological Trends, Prevention Strategies and Novel Approaches in Diagnosis and Prognosis. Community Dent. Health.

[5] Yuwanati M., Gondivkar S., Sarode S.C., Gadbail A., Desai A., Mhaske S., Pathak S.K., N Khatib M. (2021). Oral Health-Related Quality of Life in Oral Cancer Patients: Systematic Review and Meta-Analysis. Future Oncol. Lond. Engl..

[6] D’souza S., Addepalli V. (2018). Preventive Measures in Oral Cancer: An Overview. Biomed. Pharmacother..

[7] Yete S., Saranath D. (2020). MicroRNAs in Oral Cancer: Biomarkers with Clinical Potential. Oral Oncol..

[8] Warnakulasuriya S., Kujan O., Aguirre-Urizar J.M., Bagan J.V., González-Moles M.Á., Kerr A.R., Lodi G., Mello F.W., Monteiro L., Ogden G.R. (2021). Oral Potentially Malignant Disorders: A Consensus Report from an International Seminar on Nomenclature and Classification, Convened by the WHO Collaborating Centre for Oral Cancer. Oral Dis..

[9] Goldoni R., Scolaro A., Boccalari E., Dolci C., Scarano A., Inchingolo F., Ravazzani P., Muti P., Tartaglia G. (2021). Malignancies and Biosensors: A Focus on Oral Cancer Detection through Salivary Biomarkers. Biosensors.

[10] Khurshid Z., Zafar M.S., Khan R.S., Najeeb S., Slowey P.D., Rehman I.U. (2018). Role of Salivary Biomarkers in Oral Cancer Detection. Adv. Clin. Chem..

[11] Chojnowska S., Baran T., Wilińska I., Sienicka P., Cabaj-Wiater I., Knaś M. (2018). Human Saliva as a Diagnostic Material. Adv. Med. Sci..

[12] Surdu A., Foia L.G., Luchian I., Trifan D., Tatarciuc M.S., Scutariu M.M., Ciupilan C., Budala D.G. (2025). Saliva as a Diagnostic Tool for Systemic Diseases-A Narrative Review. Medicina.

[13] Nijakowski K., Owecki W., Jankowski J., Surdacka A. (2024). Salivary Biomarkers for Parkinson’s Disease: A Systematic Review with Meta-Analysis. Cells.

[14] Owecki W., Wojtowicz K., Nijakowski K. (2025). Salivary Extracellular Vesicles in Detection of Cancers Other than Head and Neck: A Systematic Review. Cells.

[15] Nijakowski K., Owecki W., Jankowski J., Surdacka A. (2024). Salivary Biomarkers for Alzheimer’s Disease: A Systematic Review with Meta-Analysis. Int. J. Mol. Sci..

[16] Nijakowski K., Zdrojewski J., Nowak M., Gruszczyński D., Knoll F., Surdacka A. (2022). Salivary Metabolomics for Systemic Cancer Diagnosis: A Systematic Review. Metabolites.

[17] Nijakowski K., Jankowski J., Gruszczyński D., Surdacka A. (2023). Salivary Alterations of Myeloperoxidase in Patients with Systemic Diseases: A Systematic Review. Int. J. Mol. Sci..

[18] Nijakowski K., Surdacka A. (2020). Salivary Biomarkers for Diagnosis of Inflammatory Bowel Diseases: A Systematic Review. Int. J. Mol. Sci..

[19] Ortarzewska M., Nijakowski K., Kolasińska J., Gruszczyński D., Ruchała M.A., Lehmann A., Surdacka A. (2023). Salivary Alterations in Autoimmune Thyroid Diseases: A Systematic Review. Int. J. Environ. Res. Public. Health.

[20] Zhang C.-Z., Cheng X.-Q., Li J.-Y., Zhang P., Yi P., Xu X., Zhou X.-D. (2016). Saliva in the Diagnosis of Diseases. Int. J. Oral Sci..

[21] Bahbah E.I., Noehammer C., Pulverer W., Jung M., Weinhaeusel A. (2021). Salivary Biomarkers in Cardiovascular Disease: An Insight into the Current Evidence. FEBS J..

[22] Owecki W., Wojtowicz K., Nijakowski K. (2025). Salivary Extracellular Vesicles in Detection of Head and Neck Cancers: A Systematic Review. Int. J. Nanomed..

[23] Bastías D., Maturana A., Marín C., Martínez R., Niklander S.E. (2024). Salivary Biomarkers for Oral Cancer Detection: An Exploratory Systematic Review. Int. J. Mol. Sci..

[24] Khijmatgar S., Yong J., Rübsamen N., Lorusso F., Rai P., Cenzato N., Gaffuri F., Del Fabbro M., Tartaglia G.M. (2024). Salivary Biomarkers for Early Detection of Oral Squamous Cell Carcinoma (OSCC) and Head/Neck Squamous Cell Carcinoma (HNSCC): A Systematic Review and Network Meta-Analysis. Jpn. Dent. Sci. Rev..

[25] Chen J., Huang Z., Chen Y., Tian H., Chai P., Shen Y., Yao Y., Xu S., Ge S., Jia R. (2025). Lactate and Lactylation in Cancer. Signal Transduct. Target. Ther..

[26] Mafessoni T.P., Mazur C.E., Amenábar J.M. (2018). Salivary Lactate Dehydrogenase (LDH) as a Tool for Early Diagnosis of Oral Cancer in Individuals with Fanconi Anemia. Med. Hypotheses.

[27] Mondal S., Adhikari N., Banerjee S., Amin S.A., Jha T. (2020). Matrix Metalloproteinase-9 (MMP-9) and Its Inhibitors in Cancer: A Minireview. Eur. J. Med. Chem..

[28] Huang H. (2018). Matrix Metalloproteinase-9 (MMP-9) as a Cancer Biomarker and MMP-9 Biosensors: Recent Advances. Sensors.

[29] St-Pierre Y. (2021). Towards a Better Understanding of the Relationships between Galectin-7, P53 and MMP-9 during Cancer Progression. Biomolecules.

[30] Treeck O., Buechler C., Ortmann O. (2019). Chemerin and Cancer. Int. J. Mol. Sci..

[31] Qi X., Fan J., Zhu J., Ling Y., Mi S., Chen H., Fan C., Li Y. (2020). Circulating Chemerin Level and Risk of Cancer: A Systematic Review and Meta-Analysis. Biomark. Med..

[32] Shin W.J., Pachynski R.K. (2018). Chemerin Modulation of Tumor Growth: Potential Clinical Applications in Cancer. Discov. Med..

[33] Al Shaar A., Hamadeh O., Ali A. (2024). Saliva and Serum Biomarkers in Oral Diseases: A Case-Control Study. Medicine.

[34] Anitha G., Kumar K.V., Deshpande G., Nagaraj M., Kalyani V. (2022). Utility of Serum and Salivary Lactate Dehydrogenase and Uric Acid Levels as a Diagnostic Profile in Oral Squamous Cell Carcinoma Patients. J. Oral Maxillofac. Pathol..

[35] Awasthi N. (2017). Role of Salivary Biomarkers in Early Detection of Oral Squamous Cell Carcinoma. Indian. J. Pathol. Microbiol..

[36] Bel’skaya L., Sarf E., Solomatin D., Kosenok V. (2020). Diagnostic and Prognostic Value of Salivary Biochemical Markers in Oral Squamous Cell Carcinoma. Diagnostics.

[37] Bhuvaneswari M., Prasad H., Rajmohan M., Sri Chinthu K.K., Prema P., Mahalakshmi L., Kumar G.S. (2022). Estimation of Salivary Lactate Dehydrogenase in Oral Squamous Cell Carcinoma, Oral Leukoplakia, and Smokers. J. Cancer Res. Ther..

[38] D’Cruz A.M., Pathiyil V. (2015). Histopathological Differentiation of Oral Squamous Cell Carcinoma and Salivary Lactate Dehydrogenase: A Biochemical Study. South Asian J. Cancer.

[39] Dhivyalakshmi M., Uma Maheswari T.N. (2014). Expression of Salivary Biomarkers-Alkaline Phosphatase & Lactate Dehydrogenase in Oral Leukoplakia. Int. J. ChemTech Res..

[40] Gholizadeh N., Alipanahi Ramandi M., Motiee-Langroudi M., Jafari M., Sharouny H., Sheykhbahaei N. (2020). Serum and Salivary Levels of Lactate Dehydrogenase in Oral Squamous Cell Carcinoma, Oral Lichen Planus and Oral Lichenoid Reaction. BMC Oral Health.

[41] Goyal G. (2020). Comparison of Salivary and Serum Alkaline Phosphates Level and Lactate Dehydrogenase Levels in Patients with Tobacco Related Oral Lesions with Healthy Subjects-A Step Towards Early Diagnosis. Asian Pac. J. Cancer Prev. APJCP.

[42] Honarmand M., Saravani R., Farhad-Mollashahi L., Smailpoor A. (2021). Salivary Lactate Dehydrogenase, c-Reactive Protein, and Cancer Antigen 125 Levels in Patients with Oral Lichen Planus and Oral Squamous Cell Carcinoma. Int. J. Cancer Manag..

[43] Joshi P.S., Golgire S. (2014). A Study of Salivary Lactate Dehydrogenase Isoenzyme Levels in Patients with Oral Leukoplakia and Squamous Cell Carcinoma by Gel Electrophoresis Method. J. Oral Maxillofac. Pathol..

[44] Kadiyala S.V. (2015). A Study of Salivary Lactate Dehydrogenase (LDH) Levels in Oral Cancer and Oral Submucosal Fibrosis Patients Amoung the Normal Individulas. J. Pharm. Sci. Res..

[45] Kallalli B.N., Rawson K., Muzammil, Singh A., Awati M.A., Shivhare P. (2016). Lactate Dehydrogenase as a Biomarker in Oral Cancer and Oral Submucous Fibrosis. J. Oral Pathol. Med..

[46] Lokesh K., Kannabiran J., Rao M.D. (2016). Salivary Lactate Dehydrogenase (LDH)—A Novel Technique in Oral Cancer Detection and Diagnosis. J. Clin. Diagn. Res. JCDR.

[47] López-Pintor R.M., González-Serrano J., Vallina C., Ivaylova Serkedzhieva K., Virto L., Nuevo P., Caponio V.C.A., Iniesta M., Rodríguez Santamarta T., Lequerica Fernández P. (2024). Factors Influencing Salivary Lactate Dehydrogenase Levels in Oral Squamous Cell Carcinoma and Oral Potentially Malignant Disorders. Front. Oral Health.

[48] Mantri T., Thete S.G., Male V., Yadav R., Grover I., Adsure G.R., Kulkarni D. (2019). Study of the Role of Salivary Lactate Dehydrogenase in Habitual Tobacco Chewers, Oral Submucous Fibrosis and Oral Cancer as a Biomarker. J. Contemp. Dent. Pract..

[49] Nandakumar E., Savitha G. (2015). A Study of Salivary Lactate Dehydragenase (LDH) Level in Normal Individuals and the Oral Cancer Patients. Res. J. Pharm. Technol..

[50] Patel S., Metgud R. (2015). Estimation of Salivary Lactate Dehydrogenase in Oral Leukoplakia and Oral Squamous Cell Carcinoma: A Biochemical Study. J. Cancer Res. Ther..

[51] Pathiyil V., D’Cruz A.M. (2017). Salivary Lactate Dehydrogenase as a Prognostic Marker in Oral Squamous Cell Carcinoma Patients Following Surgical Therapy. J. Exp. Ther. Oncol..

[52] Rathore B.S. (2024). Assessment of Salivary Biomarkers in Patients with Squamous Cell Carcinoma. Int. J. Life Sci. Biotechnol. Pharma Res..

[53] Shetty S.R., Chadha R., Babu S., Kumari S., Bhat S., Achalli S. (2012). Salivary Lactate Dehydrogenase Levels in Oral Leukoplakia and Oral Squamous Cell Carcinoma: A Biochemical and Clinicopathological Study. J. Cancer Res. Ther..

[54] Subramanian M., Fenn S., Reddy G., Rajarammohan K., Thangavelu R. (2024). Estimation of Salivary and Serum Lactate Dehydrogenase (LDH) Levels in Individuals with Oral Cancer, Oral Potentially Malignant Disorders, Tobacco Users, and Healthy Subjects-A Pilot Study. J. Indian Acad. Oral Med. Radiol..

[55] Yu J.-S., Chen Y.-T., Chiang W.-F., Hsiao Y.-C., Chu L.J., Seei L.-C., Wu C.-S., Tu H.-T., Chen H.-W., Chen C.-C. (2016). Saliva Protein Biomarkers To Detect Oral Squamous Cell Carcinoma in a High-Risk Population in Taiwan. Proc. Natl. Acad. Sci. USA.

[56] Feng Y., Li Q., Chen J., Yi P., Xu X., Fan Y., Cui B., Yu Y., Li X., Du Y. (2019). Salivary Protease Spectrum Biomarkers of Oral Cancer. Int. J. Oral Sci..

[57] Ghallab N.A., Shaker O.G. (2017). Serum and Salivary Levels of Chemerin and MMP-9 in Oral Squamous Cell Carcinoma and Oral Premalignant Lesions. Clin. Oral Investig..

[58] Krishnasree R., Jayanthi P., Varun B., Ramani P., Rathy R. (2023). Evaluation of Salivary MMP-9 in Oral Squamous Cell Carcinoma Using Enzyme Linked Immunosorbent Assay-A Pilot Study. Oral Maxillofac. Pathol. J..

[59] Nasir F.H., Mohamed R.J., Helmi R.M. (2020). Metalloproteinase Matrix of Level Saliva and Serum Carcinoma Squamous Cell. Biochem. Cell. Arch..

[60] Nisa W.-U., Khan M.A., Agha F., Khan S., Fatima S., Rathore P. (2023). Salivary Biomarkers CYFRA 21-1 and MMP9: Predictive Indicators of Disease Progression in Oral Squamous Cell Carcinoma. Pak. J. Med. Health Sci..

[61] Pazhani J., Chanthu K., Jayaraman S., Varun B.R. (2023). Evaluation of Salivary MMP-9 in Oral Squamous Cell Carcinoma and Oral Leukoplakia Using ELISA. J. Oral Maxillofac. Pathol..

[62] Peisker A., Raschke G.-F., Fahmy M.-D., Guentsch A., Roshanghias K., Hennings J., Schultze-Mosgau S. (2017). Salivary MMP-9 in the Detection of Oral Squamous Cell Carcinoma. Med. Oral Patol. Oral Cir. Bucal.

[63] Radulescu R., Totan A., Calenic B., Totan C., Greabu M. (2015). Biomarkers of Oxidative Stress, Proliferation, Inflammation and Invasivity in Saliva from Oral Cancer Patients. J. Anal. Oncol..

[64] Shin Y.-J., Vu H., Lee J.-H., Kim H.-D. (2021). Diagnostic and Prognostic Ability of Salivary MMP-9 for Oral Squamous Cell Carcinoma: A Pre-/Post-Surgery Case and Matched Control Study. PLoS ONE.

[65] Smriti K., Ray M., Chatterjee T., Shenoy R.-P., Gadicherla S., Pentapati K.-C., Rustaqi N. (2020). Salivary MMP-9 as a Biomarker for the Diagnosis of Oral Potentially Malignant Disorders and Oral Squamous Cell Carcinoma. Asian Pac. J. Cancer Prev. APJCP.

[66] Susha K.P., Ravindran R. (2023). Evaluation of Salivary Chemerin in Oral Leukoplakia, Oral Squamous Cell Carcinoma and Healthy Controls. J. Orofac. Sci..

[67] Akashanand, Zahiruddin Q.S., Jena D., Ballal S., Kumar S., Bhat M., Sharma S., Kumar M.R., Rustagi S., Gaidhane A.M. (2024). Burden of Oral Cancer and Associated Risk Factors at National and State Levels: A Systematic Analysis from the Global Burden of Disease in India, 1990–2021. Oral Oncol..

[68] OCEBM Levels of Evidence. https://www.cebm.ox.ac.uk/resources/levels-of-evidence/ocebm-levels-of-evidence.

[69] Huang L., Luo F., Deng M., Zhang J. (2024). The Relationship between Salivary Cytokines and Oral Cancer and Their Diagnostic Capability for Oral Cancer: A Systematic Review and Network Meta-Analysis. BMC Oral Health.

[70] Chiamulera M.M.A., Zancan C.B., Remor A.P., Cordeiro M.F., Gleber-Netto F.O., Baptistella A.R. (2021). Salivary Cytokines as Biomarkers of Oral Cancer: A Systematic Review and Meta-Analysis. BMC Cancer.

[71] Gallo M., Sapio L., Spina A., Naviglio D., Calogero A., Naviglio S. (2015). Lactic Dehydrogenase and Cancer: An Overview. Front. Biosci. Landmark Ed..

[72] Augoff K., Hryniewicz-Jankowska A., Tabola R. (2015). Lactate Dehydrogenase 5: An Old Friend and a New Hope in the War on Cancer. Cancer Lett..

[73] de la Cruz-López K.G., Castro-Muñoz L.J., Reyes-Hernández D.O., García-Carrancá A., Manzo-Merino J. (2019). Lactate in the Regulation of Tumor Microenvironment and Therapeutic Approaches. Front. Oncol..

[74] Comandatore A., Franczak M., Smolenski R.T., Morelli L., Peters G.J., Giovannetti E. (2022). Lactate Dehydrogenase and Its Clinical Significance in Pancreatic and Thoracic Cancers. Semin. Cancer Biol..

[75] Sharma D., Singh M., Rani R. (2022). Role of LDH in Tumor Glycolysis: Regulation of LDHA by Small Molecules for Cancer Therapeutics. Semin. Cancer Biol..

[76] Urbańska K., Orzechowski A. (2019). Unappreciated Role of LDHA and LDHB to Control Apoptosis and Autophagy in Tumor Cells. Int. J. Mol. Sci..

[77] Dong T., Liu Z., Xuan Q., Wang Z., Ma W., Zhang Q. (2017). Tumor LDH-A Expression and Serum LDH Status Are Two Metabolic Predictors for Triple Negative Breast Cancer Brain Metastasis. Sci. Rep..

[78] Wei Y., Xu H., Dai J., Peng J., Wang W., Xia L., Zhou F. (2018). Prognostic Significance of Serum Lactic Acid, Lactate Dehydrogenase, and Albumin Levels in Patients with Metastatic Colorectal Cancer. BioMed Res. Int..

[79] Zhang X., Guo M., Fan J., Lv Z., Huang Q., Han J., Wu F., Hu G., Xu J., Jin Y. (2016). Prognostic Significance of Serum LDH in Small Cell Lung Cancer: A Systematic Review with Meta-Analysis. Cancer Biomark. Sect. Dis. Markers.

[80] Cai H., Li J., Zhang Y., Liao Y., Zhu Y., Wang C., Hou J. (2019). LDHA Promotes Oral Squamous Cell Carcinoma Progression Through Facilitating Glycolysis and Epithelial-Mesenchymal Transition. Front. Oncol..

[81] Barbi W., Purohit B.M. (2022). Serum Lactate Dehydrogenase Enzyme as a Tumor Marker in Potentially Malignant Disorders: A Systematic Review and Meta-Analysis. Asian Pac. J. Cancer Prev. APJCP.

[82] Iglesias-Velázquez Ó., López-Pintor R.M., González-Serrano J., Casañas E., Torres J., Hernández G. (2022). Salivary LDH in Oral Cancer and Potentially Malignant Disorders: A Systematic Review and Meta-Analysis. Oral Dis..

[83] Quintero-Fabián S., Arreola R., Becerril-Villanueva E., Torres-Romero J.C., Arana-Argáez V., Lara-Riegos J., Ramírez-Camacho M.A., Alvarez-Sánchez M.E. (2019). Role of Matrix Metalloproteinases in Angiogenesis and Cancer. Front. Oncol..

[84] Choi E.K., Kim H.D., Park E.J., Song S.Y., Phan T.T., Nam M., Kim M., Kim D.-U., Hoe K.-L. (2023). 8-Methoxypsoralen Induces Apoptosis by Upregulating P53 and Inhibits Metastasis by Downregulating MMP-2 and MMP-9 in Human Gastric Cancer Cells. Biomol. Ther..

[85] Yin P., Su Y., Chen S., Wen J., Gao F., Wu Y., Zhang X. (2021). MMP-9 Knockdown Inhibits Oral Squamous Cell Carcinoma Lymph Node Metastasis in the Nude Mouse Tongue-Xenografted Model through the RhoC/Src Pathway. Anal. Cell. Pathol. Amst..

[86] Li Y., He J., Wang F., Wang X., Yang F., Zhao C., Feng C., Li T. (2020). Role of MMP-9 in Epithelial-Mesenchymal Transition of Thyroid Cancer. World J. Surg. Oncol..

[87] Patterson B.C., Sang Q.A. (1997). Angiostatin-Converting Enzyme Activities of Human Matrilysin (MMP-7) and Gelatinase B/Type IV Collagenase (MMP-9). J. Biol. Chem..

[88] Vilen S.-T., Salo T., Sorsa T., Nyberg P. (2013). Fluctuating Roles of Matrix Metalloproteinase-9 in Oral Squamous Cell Carcinoma. Sci. World J..

[89] Zheng W.-Y., Zhang D.-T., Yang S.-Y., Li H. (2015). Elevated Matrix Metalloproteinase-9 Expression Correlates With Advanced Stages of Oral Cancer and Is Linked to Poor Clinical Outcomes. J. Oral Maxillofac. Surg..

[90] Deng W., Peng W., Wang T., Chen J., Zhu S. (2019). Overexpression of MMPs Functions as a Prognostic Biomarker for Oral Cancer Patients: A Systematic Review and Meta-Analysis. Oral Health Prev. Dent..

[91] Bozaoglu K., Bolton K., McMillan J., Zimmet P., Jowett J., Collier G., Walder K., Segal D. (2007). Chemerin Is a Novel Adipokine Associated with Obesity and Metabolic Syndrome. Endocrinology.

[92] Goralski K.B., Jackson A.E., McKeown B.T., Sinal C.J. (2019). More Than an Adipokine: The Complex Roles of Chemerin Signaling in Cancer. Int. J. Mol. Sci..

[93] Buechler C., Feder S., Haberl E.M., Aslanidis C. (2019). Chemerin Isoforms and Activity in Obesity. Int. J. Mol. Sci..

[94] Treeck O., Buechler C. (2020). Chemerin Signaling in Cancer. Cancers.

[95] Wang N., Wang Q.-J., Feng Y.-Y., Shang W., Cai M. (2014). Overexpression of Chemerin Was Associated with Tumor Angiogenesis and Poor Clinical Outcome in Squamous Cell Carcinoma of the Oral Tongue. Clin. Oral Investig..

[96] Lu Z., Liang J., He Q., Wan Q., Hou J., Lian K., Wang A. (2019). The Serum Biomarker Chemerin Promotes Tumorigenesis and Metastasis in Oral Squamous Cell Carcinoma. Clin. Sci..

[97] Gao F., Feng Y., Hu X., Zhang X., Li T., Wang Y., Ge S., Wang C., Chi J., Tan X. (2023). Neutrophils Regulate Tumor Angiogenesis in Oral Squamous Cell Carcinoma and the Role of Chemerin. Int. Immunopharmacol..

[98] Hu X., Wang N., Gao F., Ge S., Lin M., Zhang X., Li T., Li T., Xu C., Huang C. (2024). Prognostic Significance of Serum Chemerin and Neutrophils Levels in Patients with Oral Squamous Cell Carcinoma. Heliyon.

[99] Lu Z., Liu J., Wan Q., Wu Y., Wu W., Chen Y. (2024). Chemerin Promotes Invasion of Oral Squamous Cell Carcinoma by Stimulating IL-6 and TNF-α Production via STAT3 Activation. Mol. Biol. Rep..

[100] Page M.J., McKenzie J.E., Bossuyt P.M., Boutron I., Hoffmann T.C., Mulrow C.D., Shamseer L., Tetzlaff J.M., Akl E.A., Brennan S.E. (2021). The PRISMA 2020 Statement: An Updated Guideline for Reporting Systematic Reviews. BMJ.

[101] Study Quality Assessment Tools|NHLBI, NIH. https://www.nhlbi.nih.gov/health-topics/study-quality-assessment-tools.

