# Supplementary material for: Salivary Lactate Dehydrogenase, Matrix Metalloproteinase-9, and Chemerin—The Most Promising Biomarkers for Oral Cancer? A Systematic Review with Meta-Analysis
Source: Int J Mol Sci. 2025 Aug 18;26(16):7947. doi: 10.3390/ijms26167947 (PMC12386804; doi:10.3390/ijms26167947)
Supplement: Supplementary file 1 [file ijms-26-07947-s001.zip › ijms-3752490-supplementary.pdf]

	clearly stated research question or objective	clearly defined study population	sample size justification	groups recruitment from the same population	valid inclusion and exclusion criteria	cases differentiated from controls	randomization	clearly defined measures	blinded status of participants	adjusted statistical methods	summarized quality score
Al Shaar et al., 2024	🟢	🟡	🟡	🟢	🟢	🟢	🟡	🟢	🟡	🟢	🟢
Anitha et al., 2022	🟢	🟢	🟢	🟡	🟢	🟢	🟡	🟢	🟡	🟢	🟢
Awasthi et al., 2017	🟢	🟢	🟡	🟢	🟢	🟢	🟡	🟢	🟡	🟢	🟢
Bel'skaya et al., 2020	🟢	🔴	🔴	🟡	🟢	🟢	🟡	🟢	🟡	🟢	🟡
Bhuvaneswari et al., 2022	🟢	🔴	🟡	🟡	🟢	🟢	🟡	🟢	🟡	🟢	🟡
D'Cruz et al., 2015	🟢	🟡	🟢	🟢	🟢	🟢	🟡	🟡	🟡	🟢	🟢
Dhivyalakshmi et al., 2014	🟡	🔴	🟢	🟡	🟢	🟢	🟡	🟢	🟡	🟢	🟡
Feng et al., 2019	🟢	🔴	🟢	🟡	🟢	🟢	🟡	🟢	🟡	🟢	🟢
Ghallab et al., 2017	🟢	🟡	🟢	🟢	🟢	🟢	🟡	🟢	🟡	🟢	🟢
Gholizadeh et al., 2020	🟢	🟡	🟢	🟢	🟢	🟢	🟡	🟢	🟡	🟢	🟢
Goyal et al., 2020	🟢	🔴	🟢	🟢	🟢	🟢	🟡	🟢	🟡	🟢	🟢
Honarmand et al., 2021	🟢	🟡	🟡	🟢	🟢	🟢	🟡	🟢	🟡	🟢	🟢
Joshi et al., 2014	🟢	🟡	🟢	🟡	🟢	🟢	🟡	🟢	🟡	🟢	🟢
Kadiyala et al., 2015	🟢	🔴	🟢	🟡	🟢	🟢	🟡	🟢	🟡	🟢	🟢
Kallalli et al., 2016	🟢	🔴	🔴	🟡	🟢	🟢	🟡	🟡	🟡	🟢	🟡
Krishnasree et al., 2023	🟢	🟡	🟢	🟡	🟢	🟢	🟡	🟡	🟡	🟢	🟢
Lokesh et al., 2016	🟢	🟡	🔴	🟡	🟢	🟢	🟡	🟡	🟡	🟢	🟡
López-Pintor et al., 2024	🟢	🟢	🟡	🟢	🟢	🟢	🟡	🟢	🟡	🟢	🟢
Mantri et al., 2019	🟢	🔴	🟢	🟢	🟢	🟢	🟡	🟢	🟡	🟢	🟢
Nandakumar et al., 2015	🟡	🟡	🟢	🟡	🟢	🟢	🟡	🟢	🟡	🟢	🟢
Nasir et al., 2020	🟢	🔴	🟢	🟡	🟡	🟢	🟡	🟡	🟡	🟢	🟡
Nisa et al., 2023	🟢	🟡	🟢	🟢	🟢	🟢	🟡	🟢	🟡	🟢	🟢
Patel et al., 2015	🟢	🟡	🟢	🟢	🟢	🟢	🟡	🟡	🟡	🟢	🟢
Pathiyil et al., 2017	🟢	🔴	🟢	🟢	🟢	🟢	🟡	🟢	🟡	🟢	🟢
Pazhani et al., 2023	🟢	🟢	🟢	🟡	🟢	🟢	🟡	🟡	🟡	🟢	🟢
Peisker et al., 2017	🟢	🟢	🟢	🟢	🟢	🟢	🟡	🟢	🟡	🟢	🟢
Radulescu et al., 2015	🟢	🟡	🔴	🟡	🟢	🟢	🟡	🟢	🟡	🟢	🟡
Rathore et al., 2024	🟢	🟡	🟢	🟡	🟡	🟢	🟡	🟡	🟡	🟢	🟡
Shetty et al., 2012	🟢	🔴	🟢	🟢	🟢	🟢	🟡	🟢	🟡	🟢	🟢
Shin et al., 2021	🟢	🟡	🟡	🟢	🟢	🟢	🟡	🟢	🟡	🟢	🟢
Smriti et al., 2020	🟢	🟢	🔴	🟡	🟢	🟢	🟡	🟢	🟡	🟢	🟢
Subramanian et al., 2024	🟢	🟡	🟢	🟡	🟢	🟢	🟢	🟢	🟡	🟢	🟢
Susha et al., 2023	🟢	🟡	🟢	🟢	🟢	🟢	🟡	🟢	🟡	🟢	🟢
Yu et al., 2016	🟢	🟡	🟡	🟡	🟢	🟢	🟢	🟢	🟢	🟢	🟢

Figure S1. Quality assessment of the included studies.

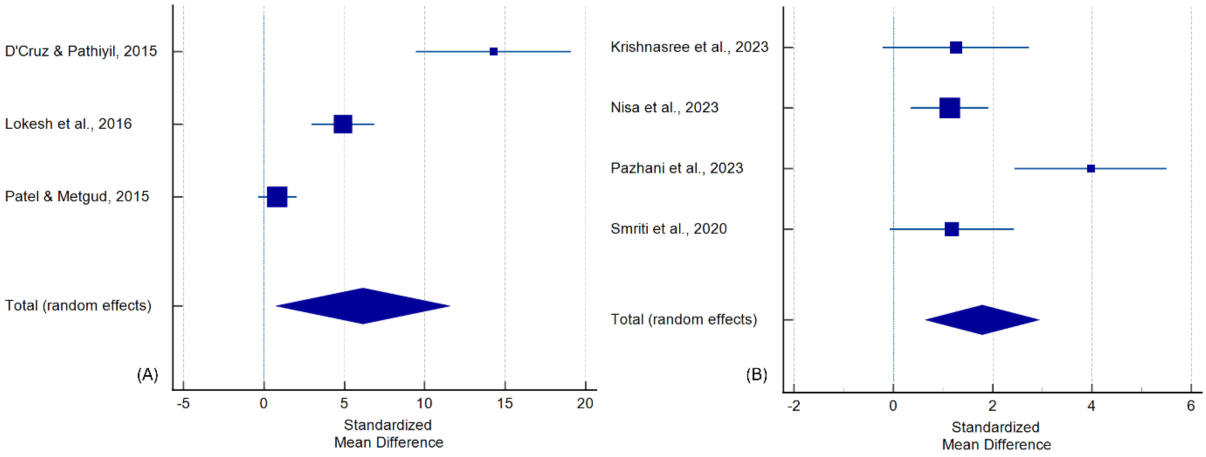

Figure S2. Forest plot with standardized mean differences comparing levels between poorly and well-differentiated OC patients for (A) LDH, (B) MMP-9.

Owecki, W.; Nijakowski, K. Salivary Lactate Dehydrogenase, Matrix Metalloproteinase-9, and Chemerin—The Most Promising Biomarkers for Oral Cancer? A Systematic Review with Meta-Analysis
